# Cardiac output estimation: Vigileo and Mostcare versus echocardiography

**DOI:** 10.1186/cc12135

**Published:** 2013-03-19

**Authors:** S Romagnoli, D Quattrone, AR De Gaudio

**Affiliations:** 1 Azienda Ospedaliero Universitaria Careggi, Florence, Italy

## Introduction

In the present study we analyzed the reliability for cardiac output (CO) measurement of Vigileo (Edwards Lifescience, Irvine, CA, USA) and MostCare (pressure recording analytical method; Vygon-Vytech, Padova, Italy) in comparison with transthoracic Doppler echocardiography (as the reference method) in patients undergoing vascular surgery.

## Methods

Both Vigileo and MostCare were connected to the FloTrac transducer (Edwards Lifescience) for CO calculation. The data from Vigileo and MostCare were registered (COVIG and COMC respectively) and compared with those calculated with the echocardiographic standard formulation (stroke volume = cross-sectional area×velocity time integral; COECHO = SV×heart rate). In every patient CO was measured twice: at baseline (T1) and after volume loading (500 ml lactate Ringer solution) (T2). Agreements between COVIG, COMC, and COECHO were evaluated by means of simple linear regression (*r*^2^) and Bland-Altman analysis.

## Results

**Figure 1:**
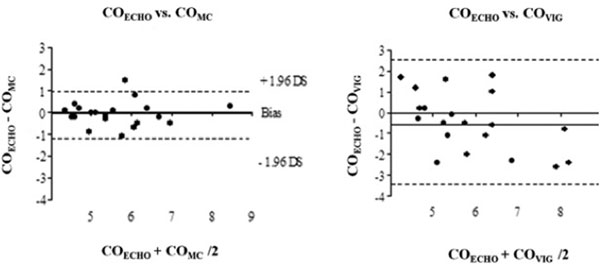
**Bland-Altman analysis (T1)**.

Twenty patients were enrolled in the study. Values of *r*^2^, bias and limit of agreement at T1 and T2 are summarized in Table 1. CO values ranged from 3.9 and 8.6 l/minute (echo), from 3.4 to 9.9 (Vigileo) and from 4 to 8.3 (MostCare); the Pearson's and Bland-Altman methods showed poor agreement between COECHO and COVIG, demonstrating a tendency to overestimation (see Figure 1). The percentage of error (PE) was 51.7% at T1 and 49.3% at T2. On the contrary, MostCare measures showed good agreement with echocardiography (see Table 1) with a PE of 22.4% at T1 and of 17% at T2.

**Table 1:** 

	Pearson *r*^2^	Bias	LoA
COECHO vs. COVIG (T1)	0.31 (CI 0.24 to 1.63)	-0.49	-3.41 to 2.43
COECHO vs. COMC (T1)	0.71 (CI 0.56 to 1.08)	-0.055	-1.19 to 1.08
COECHO vs. COVIG (T2)	0.27 (CI 0.16 to 1.53)	-0.49	-3.24 to 2.34
COECHO vs. COMC (T2)	0.79 (CI 0.66 to 1.107)	-0.023	-0.94 to 0.9

CI, confidence interval; LoA, limits of agreement.

## Conclusion

Vigileo did not prove to be a substitute to the reference system; pre-loaded data, necessary for vascular impedance estimation, may be one of the main limitations that made Vigileo measurements less accurate than the MostCare ones. On the contrary, MostCare, an uncalibrated totally independent system, was shown to properly estimate the vascular impedance in these hemodynamically stable patients. Further comparisons in unstable conditions are needed to confirm our observations.
